# Development and validation of a clinical prediction model for poor ovarian response in assisted reproductive technology

**DOI:** 10.3389/fendo.2026.1732869

**Published:** 2026-06-15

**Authors:** Xin Xin, Zhaoxia Cheng, Ting Hu, Yi Guo, Nan Li, Junbo Zhao, Shuaishuai Guo

**Affiliations:** 1 Department of Reproductive Medicine, Shenyang Women’s and Children’s Hospital, Shenyang, Liaoning, China; 2 Liaoning Key Laboratory of Assisted Reproduction, Shenyang, Liaoning, China; 3 Shenyang Clinical Medical Research Center for Assisted Reproduction, Shenyang, Liaoning, China

**Keywords:** assisted reproductive technology, clinical prediction model, nomogram, ovarian reserve markers, poor ovarian response

## Abstract

**Objective:**

The aim of this study was to develop and validate a predictive model for poor ovarian response (POR) in patients undergoing Assisted Reproductive Technology (ART).

**Methods:**

This retrospective cohort study included 1,789 patients who underwent IVF/ICSI with a GnRH antagonist protocol at the reproductive medicine center of Shenyang Women’s and Children’s Hospital between January 2020 and December 2023. Data from January 2020 to April 2023 were used for model development, and data from May 2023 to December 2023 was used for validation. Clinical and ovarian reserve markers were collected. Four prediction models were developed and compared: (1) logistic regression with stepwise selection, (2) full-variable logistic regression, (3) LASSO regression with the minimum lambda (λ_min) criterion, and (4) LASSO regression with the 1-standard error (1-SE) rule. The optimal model was selected based on discrimination (area under the receiver operating characteristic curve, AUC), calibration, and Brier score.

**Results:**

The final model, based on stepwise logistic regression, identified AMH, basal FSH, BMI, and antral follicle count as significant predictors of POR. The model exhibited high discriminatory ability and good calibration across the training, internal validation, and test datasets. The nomogram developed from this model provides an easy-to-use tool for individualized risk prediction, enabling clinicians to optimize treatment strategies for patients at risk of POR.

**Conclusion:**

This study provides a comprehensive predictive model for POR in ART, incorporating a wide range of clinical and ovarian reserve markers. The stepwise logistic regression model offers superior predictive accuracy and clinical utility, making it a valuable tool for personalized ART management. The nomogram developed enhances decision-making in ART by offering an intuitive, evidence-based method for predicting POR risk.

## 1 Introduction

Assisted Reproductive Technology (ART) has become a cornerstone in the management of infertility, and ovarian response to gonadotropin stimulation is one of the most critical factors determining ART success. However, a subset of women undergoing ART experience poor ovarian response (POR), characterized by insufficient follicular development despite undergoing standard ovarian stimulation protocols. POR leads to significantly lower oocyte retrieval numbers, increases the likelihood of cycle cancellation, reduces the chances of successful embryo transfer, and escalates both the economic and psychological burden for patients (1). Despite the development of diagnostic criteria such as the Bologna criteria, which defines POR as fewer than three oocytes retrieved during ovarian stimulation, and the POSEIDON classification, which aims to stratify patients based on ovarian reserve and age, the ability to predict poor ovarian response before ART treatment remains limited (2, 3). The POSEIDON criteria provide a framework for better categorizing patients with poor ovarian reserve; however, these standards are primarily used for diagnostic purposes rather than predictive modeling, which is essential for guiding treatment decisions (4). Currently, clinicians lack a systematic, quantitative, and prospective clinical prediction model to identify women at risk for POR before ovarian stimulation begins, preventing early intervention and individualized strategy adjustments.

Existing research on POR prediction typically focuses on ovarian reserve markers, such as Anti-Müllerian Hormone (AMH), Antral Follicle Count (AFC), and basal FSH levels (5, 6). Although these markers provide valuable insight into the ovarian reserve, their predictive value is often limited, particularly when considered in isolation. Furthermore, these markers do not fully capture the complexity of ovarian response, as they fail to account for factors like age, medical history, and BMI (7, 8). For instance, increased BMI has been shown to negatively affect ovarian function and response to stimulation by disrupting hormonal regulation and causing insulin resistance, which are both known to impair follicular development and oocyte quality (9). Despite the evidence linking these factors to POR, most existing models for predicting poor ovarian response do not adequately incorporate such clinical variables, leading to limited generalizability and predictive performance. Advanced statistical techniques, including LASSO regression, will be utilized for feature selection and model construction. LASSO regression is a powerful tool for preventing overfitting and identifying key predictors from a large set of clinical variables, thus ensuring that the model is both efficient and interpretable (10).

This study aims to fill this gap by developing a comprehensive, real-world predictive model for POR. By incorporating a broad range of clinical and biological variables, this model aims to more accurately predict which patients are at risk for POR before initiating ART treatment, thereby enabling clinicians to adjust stimulation protocols accordingly.

## 2 Methods

### 2.1 Study design and population

This study was conducted to develop and validate a clinical prediction model for Poor Ovarian Response (POR) in patients undergoing assisted reproductive technology (ART). According to the Bologna criteria (11) and Poseidon criteria (12), poor ovarian response (POR) in this study is defined as either a cycle in which oocyte retrieval is canceled due to poor ovarian response or a cycle where the number of oocytes retrieved is ≤ 3. All cases with cycles canceled due to poor ovarian response were retained in the analysis, coded as having “0 oocytes retrieved,” and were not excluded by the study design. A retrospective cohort was established, and the dataset was divided into a training set, an internal validation set, and a test set to ensure robust model performance assessment. Only one stimulation cycle per woman was included in the analysis; if multiple cycles were available, only the first cycle was used to ensure that all observations were independent. All data were collected during the 2nd to 4th days of the menstrual cycle, prior to controlled ovarian hyperstimulation (COH).

Comprehensive baseline characteristics were collected, including female age, infertility duration, ART indication (primary or secondary infertility), body mass index (BMI), baseline levels of follicle-stimulating hormone (FSH), luteinizing hormone (LH), estradiol (E2), antral follicle count (AFC), and anti-Müllerian hormone (AMH), and relevant medical history variables, including previous ovarian surgery and pelvic inflammatory disease. A total of 1,789 women undergoing antagonist protocols were included. This study included 1,486 patients from the Reproductive Medicine Center of Shenyang Women’s and Children’s Hospital between January 2020 and April 2023, who comprised the development dataset. These patients were randomly divided into a training set (n = 1,042) and an internal validation set (n = 444). A test dataset of 303 patients treated at the same center between May 2023 and December 2023 was collected for model performance evaluation.

### 2.2 Inclusion and exclusion criteria

Inclusion Criteria: (1) IVF/ICSI patients undergoing antagonist protocol stimulation;(2) Complete electronic medical records, including all relevant clinical parameters and treatment information.; (3) Complete ovarian function assessment records, including baseline levels of FSH, LH, E2, and AMH;(4) At least one initiated ovarian stimulation cycle with corresponding treatment and outcome records (including cycles canceled for any reason). Exclusion Criteria: (1) Patients with more than 15 oocytes retrieved; (2) Patients with other systemic cancers, malignant diseases, or uncontrolled or acute conditions deemed ART contraindications after clinical evaluation; (3) Egg donation cycles, as patients undergoing egg donation have significantly different ovarian function compared to the general population; (4) Patients with significant endocrine disorders or abnormal hormone levels that may interfere with the study outcomes; (5) Patients enrolled in other clinical trials that may introduce potential confounding factors; (6) Patients with missing key clinical variables.

### 2.3 Predictor selection and model development

Candidate predictors were selected based on clinical relevance and prior literature. To optimize variable selection and prevent overfitting, Least Absolute Shrinkage and Selection Operator (LASSO) regression was applied, which imposes an L1 penalty to shrink coefficients of less informative variables to zero (13). For comparison, stepwise selection Logistic regression (14) and a full-variable Logistic regression model was also developed. Importantly, only baseline variables available before the initiation of ovarian stimulation were considered as candidate predictors. These variables were retained only for descriptive purposes and were not interpreted inferentially. The final models included:

1. Logistic regression with stepwise selection, which iteratively selects predictors based on statistical significance;
2. Full-variable Logistic regression model, incorporating all available predictors without selection constraints;
3. A LASSO regression model employing the minimum lambda (λ_min) criterion, which selects the penalty parameter that yields the minimum cross-validation error.
4. A more parsimonious LASSO regression model selected via the 1-standard error (1-SE) rule. This approach chooses the largest λ within one standard error of λ_min, favoring a simpler model while maintaining comparable predictive performance.

### 2.4 Model performance evaluation

The predictive performance of each model was assessed in terms of discrimination, calibration, and clinical utility. The receiver operating characteristic (ROC) curve and its corresponding area under the curve (AUC) were used to assess the model’s discrimination—that is, its ability to distinguish between patients with and without POR (15). Calibration curves, accompanied by the intercept and slope, were plotted to evaluate calibration, which quantifies the agreement between the predicted probabilities and the actual observed outcomes; a well-calibrated model demonstrates predictions that align closely with event rates across risk strata. The Brier score was calculated as a composite measure of overall prediction accuracy, incorporating both discrimination and calibration, with lower values indicating superior predictive performance.

### 2.5 Model validation

Model validation followed a predefined and non-overlapping data-splitting strategy. In the model validation process, transparent data partitioning was implemented. The development dataset (n = 1,486), collected from the Reproductive Medicine Center of Shenyang Women’s and Children’s Hospital between January 2020 and April 2023, was randomly split by patient ID in a 7:3 ratio using the R caret package, with the random seed fixed at 225, resulting in a training subset (n = 1,042) and an internal validation subset (n = 444). A test dataset (n = 303), collected from same center (May 2023–December 2023), was used for final model evaluation. A 10-fold cross-validation was applied to the training set to guide the LASSO variable selection. This process selected the regularization parameter (λ) that minimized the cross-validation error, rigorously confining the selection step to the training data. Neither the internal validation subset nor the test cohort was used during model training or cross-validation. Performance metrics—including AUC, calibration slope, and Brier score—were independently evaluated in the internal validation subset and the test cohort to assess discrimination and calibration. The DeLong test was used to compare the area under the curve (AUC) among different models.

### 2.6 Nomogram development

A nomogram was constructed based on the best-performing model to facilitate individualized risk prediction and enhance clinical usability (16). This graphical tool, which combines multiple clinical and demographic predictors, provides an intuitive and quantitative method for estimating the probability of poor ovarian response (POR) in individual patients. The nomogram assigns a specific score to each predictor, with the total score corresponding to the estimated POR probability. This allows clinicians to quickly assess patient risk, make informed treatment decisions, and tailor interventions accordingly. The development of the nomogram involved careful calibration of the model, ensuring that predicted probabilities aligned with observed outcomes.

### 2.7 Statistical analysis

All statistical analyses were conducted using R software (version 4.3, R Foundation for Statistical Computing, Vienna, Austria) (17). The glmnet package was used for LASSO regression (18), while rms and pROC were employed for calibration and ROC analyses. Continuous variables were compared using Student’s t-test or Mann–Whitney U test, as appropriate, while categorical variables were analyzed using chi-square or Fisher’s exact test (19). A two-sided p-value <0.05 was considered statistically significant.

### 2.8 Missing data handling

To ensure robust model development and transparent reporting, a systematic approach was adopted for handling missing data. First, the proportion of missing values was calculated for all patients within the pooled analysis dataset intended for model development. Variables exhibiting a missing rate exceeding 10% were considered to have substantial missingness and were consequently excluded from the predictive model. For all remaining variables with a missing rate of 10% or less, missing values were imputed within this pooled analysis set: continuous variables were imputed with the median value, and categorical variables were imputed with the overall mode. It is noted that the reference to a complete-case analysis in Exclusion Criterion 6 pertained strictly to the initial cohort derivation and descriptive phase; the final modeling cohort applied the imputation strategy described above to preserve sample size. Comprehensive reporting of missing data patterns, including a participant flow diagram and a detailed missingness table stratified by dataset and outcome, is provided in the Results section.

## 3 Result

### 3.1 Baseline characteristics

**Figure 1:**
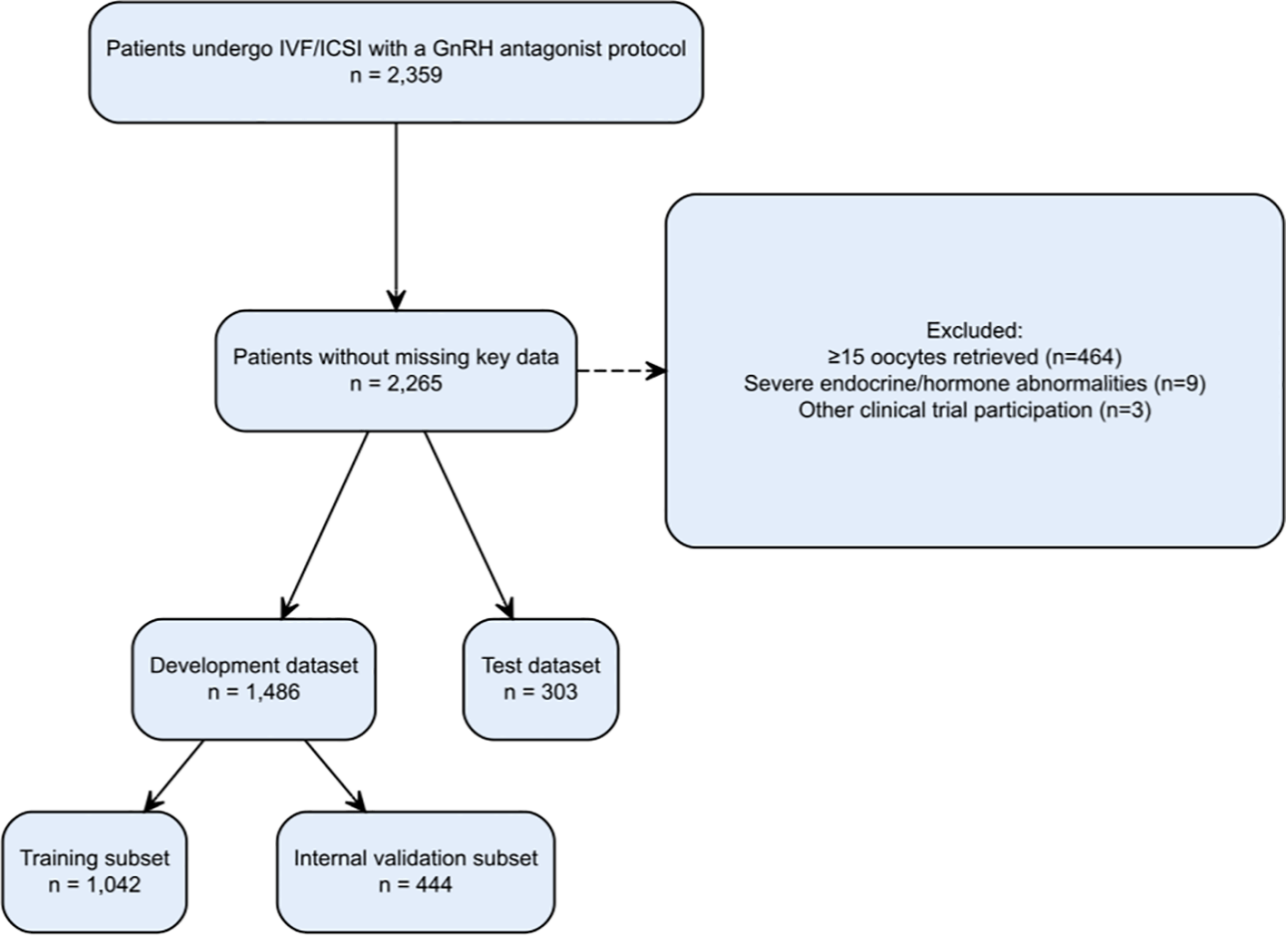
Flowchart of patient enrollment and group allocation.

A total of 1,789 patients were included from reproductive medicine center of Shenyang Women’s and Children’s Hospital, with 1324 (89.10%) in the normal ovarian response group and 162 (10.90%) in the poor ovarian response (POR) group in development dataset and with 276 (91.09%) in the normal ovarian response group and 27 (8.91%) in the poor ovarian response (POR) group in test dataset (**Figure 1**). In the development dataset,the POR group was significantly older (35.23 ± 4.72 vs. 31.72 ± 3.85 years, P < 0.0001), had a higher BMI (24.82 ± 3.41 vs. 23.72 ± 3.74, P = 0.0004) and Basal FSH (8.75 ± 2.33 vs. 6.59 ± 1.58, P < 0.0001). Ovarian reserve markers, including AMH, Basal LH and Antral Follicle Count, were significantly lower in the POR group (P < 0.05). Additionally, a history of ovarian surgery and pelvic inflammatory disease was more common in the POR group (P < 0.05) (**Table 1**). Overall, the baseline characteristics were well-balanced between the training and validation sets (**Supplementary Table 2**).

**Table 1:** Baseline characteristics of patients with normal and poor ovarian response.

Item	Category	Total cohort	Normal ovarian response	Poor ovarian response	P-value	statistic
Female Age		32.10 ± 4.10	31.72 ± 3.85	35.23 ± 4.72	<0.0001	-10.67
Duration of Infertility		3.85 ± 2.73	3.82 ± 2.69	4.08 ± 3.03	0.2485	-1.15
ART-Indicated Infertility	Primary Infertility	814 (54.78%)	737 (55.66%)	77 (47.53%)	0.0496	3.85
	Secondary Infertility	672 (45.22%)	587 (44.34%)	85 (52.47%)		
	Total	1486 (100%)	1324 (100%)	162 (100%)		
BMI		23.84 ± 3.72	23.72 ± 3.74	24.82 ± 3.41	0.0004	-3.58
Basal FSH		6.83 ± 1.81	6.59 ± 1.58	8.75 ± 2.33	<0.0001	-15.41
Basal LH		4.96 ± 2.31	5.01 ± 2.32	4.52 ± 2.24	0.011	2.55
Basal E		35.68 ± 14.98	35.42 ± 14.70	37.86 ± 16.99	0.0501	-1.96
Antral Follicle Count		13.14 ± 4.80	13.98 ± 4.34	6.29 ± 2.10	<0.0001	22.23
AMH		3.08 ± 0.37	3.14 ± 0.34	2.60 ± 0.28	<.0001	19.61
Ovarian Surgery History	None	1465 (98.59%)	1314 (99.24%)	151 (93.21%)	<0.0001	37.73
	Yes	21 (1.41%)	10 (0.76%)	11 (6.79%)		
	Total	1486 (100%)	1324 (100%)	162 (100%)		
History of Pelvic Inflammation	None	1468 (98.79%)	1311 (99.02%)	157 (96.91%)	0.0208	5.34
	Yes	18 (1.21%)	13 (0.98%)	5 (3.09%)		
	Total	1486 (100%)	1324 (100%)	162 (100%)		

### 3.2 Data missingness and imputation

The overall data quality was high, with a low proportion of missing values across the analyzed variables. As detailed in **Supplementary Table 1**, only basal FSH levels were missing in a small subset of participants, accounting for approximately 0.29% of the training dataset, 0.23% of the internal validation dataset, and 0.00% of the test dataset. These missing values were imputed using the median value of 6.65. No other variables contained missing data exceeding the predefined threshold of 10%, and thus no further variables were excluded on the basis of missingness.

### 3.3 Cross-validation and variable selection pathway analysis

To further refine our predictive model for poor ovarian response, we conducted cross-validation and variable selection pathway analysis, as illustrated in **Supplementary Figure**. The cross-validation curve (**Supplementary Figure 1**) displays the binomial deviance across different values of log(λ), helping identify the optimal regularization parameter in the LASSO regression model. The lowest binomial deviance corresponds to the most suitable λ value, ensuring a balance between model complexity and predictive performance. As λ decreases, the model retains more variables, gradually improving fit but potentially increasing overfitting risk. The variable selection pathway (**Supplementary Figure 2**) demonstrates how coefficients of different predictors evolve as λ changes. As regularization strength increases (higher λ values), non-essential variables shrink toward zero, leaving only the most influential predictors. This visualization confirms that a subset of key variables consistently contributes to predicting poor ovarian response, reinforcing their robustness across different modeling strategies.

### 3.4 Comparison of prediction models for poor ovarian response

To predict poor ovarian response, we applied four modeling approaches: stepwise logistic regression, full-variable logistic regression, post-LASSO refit logistic regression model (minimum lambda), and post-LASSO refit logistic regression model (one standard error of minimum lambda).

Stepwise logistic regression (**Table 2**) identified AMH, Basal FSH, BMI, and antral follicle count as significant predictors (P<0.05). In the full-variable logistic regression (**Supplementary Table 3**), after adjusting for all covariates, basal FSH and BMI were identified as risk factors, while antral follicle count and AMH were protective factors for the outcome.

**Table 2:** Stepwise logistic regression for predicting poor ovarian response.

Item	Univariate OR (95% CI)	Univariate P-value	Multivariate OR (95% CI)	Multivariate P-value
AMH	0.00(0.00-0.01)	<.0001	0.11(0.04-0.34)	<.0001
Basal FSH	1.94(1.71-2.19)	<.0001	1.32(1.13-1.54)	0.0004
BMI	1.07(1.02-1.12)	0.0062	1.12(1.03-1.22)	0.0073
Antral Follicle Count	0.44(0.39-0.51)	<.0001	0.51(0.45-0.59)	<.0001
Age	1.22(1.16-1.28)	<.0001		.
ART-Indicated Infertility	1.70(1.16-2.49)	0.0067		.
Basal E	1.01(1.00-1.02)	0.1327		.
Basal LH	0.85(0.77-0.95)	0.0031		.
Duration of Infertility	1.01(0.94-1.08)	0.755		.
History of Pelvic Inflammation	3.32(0.85-13.01)	0.0851		.
Ovarian Surgery History	12.97(4.17-40.32)	<.0001		.

The Post-LASSO refit logistic regression model (using the one standard error of minimum lambda criterion) retained age, AMH, basal FSH, BMI, antral follicle count, and history of ovarian surgery as predictor variables (**Supplementary Table 4**), although age and history of ovarian surgery did not reach statistical significance in the final logistic regression model. This model highlighted the importance of ovarian reserve markers (AMH) in predicting poor ovarian response. The Post-LASSO Refit Logistic Regression (Minimum Lambda) Model (**Supplementary Table 5**) excluded few variables, with all except ART-Indicated Infertility being retained in the final model. Notably, Basal FSH had a strong association with poor ovarian response in this model, while AMH continued to demonstrate a protective effect against the outcome.

Across all models, higher antral follicle count and AMH consistently demonstrated protective effects, while higher basal FSH and BMI were associated with an increased risk of poor ovarian response. The consistency of these variables across different modeling approaches highlights their potential as key predictors for ovarian response classification. These findings suggest that integrating multiple factors, including hormonal markers and ovarian reserve indicators, may enhance the prediction of ovarian response in assisted reproductive technology (ART) settings.

### 3.5 Comparison of discriminatory performance using ROC curve analysis

**Figure 2:**
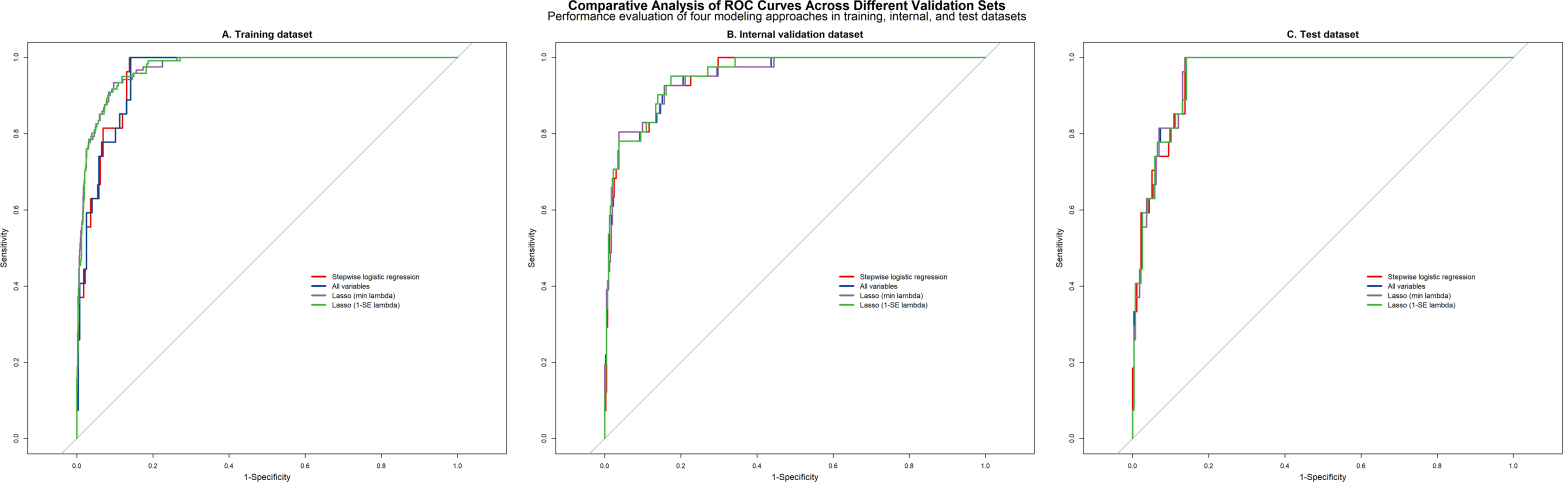
Comparative Analysis of ROC Curves Across Different Validation Sets. **(A)** Training dataset; **(B)** Internal validation dataset; **(C)** Test dataset.

The predictive performance of different models was evaluated using receiver operating characteristic (ROC) curves, with the area under the curve (AUC) as the primary measure of discrimination. In the training set, all models exhibited strong predictive power, indicating high sensitivity and specificity in distinguishing patients with Poor Ovarian Response (POR). This strong performance was maintained in the internal validation set and the test set. All models demonstrated high discriminatory performance, and since comparisons of AUCs between models showed no statistically significant differences, no single model could be selected based on discrimination metrics alone (**Figure 2**, **Supplementary Table 6**).

### 3.6 Assessment of model calibration using calibration curves and Brier score

**Figure 3:**
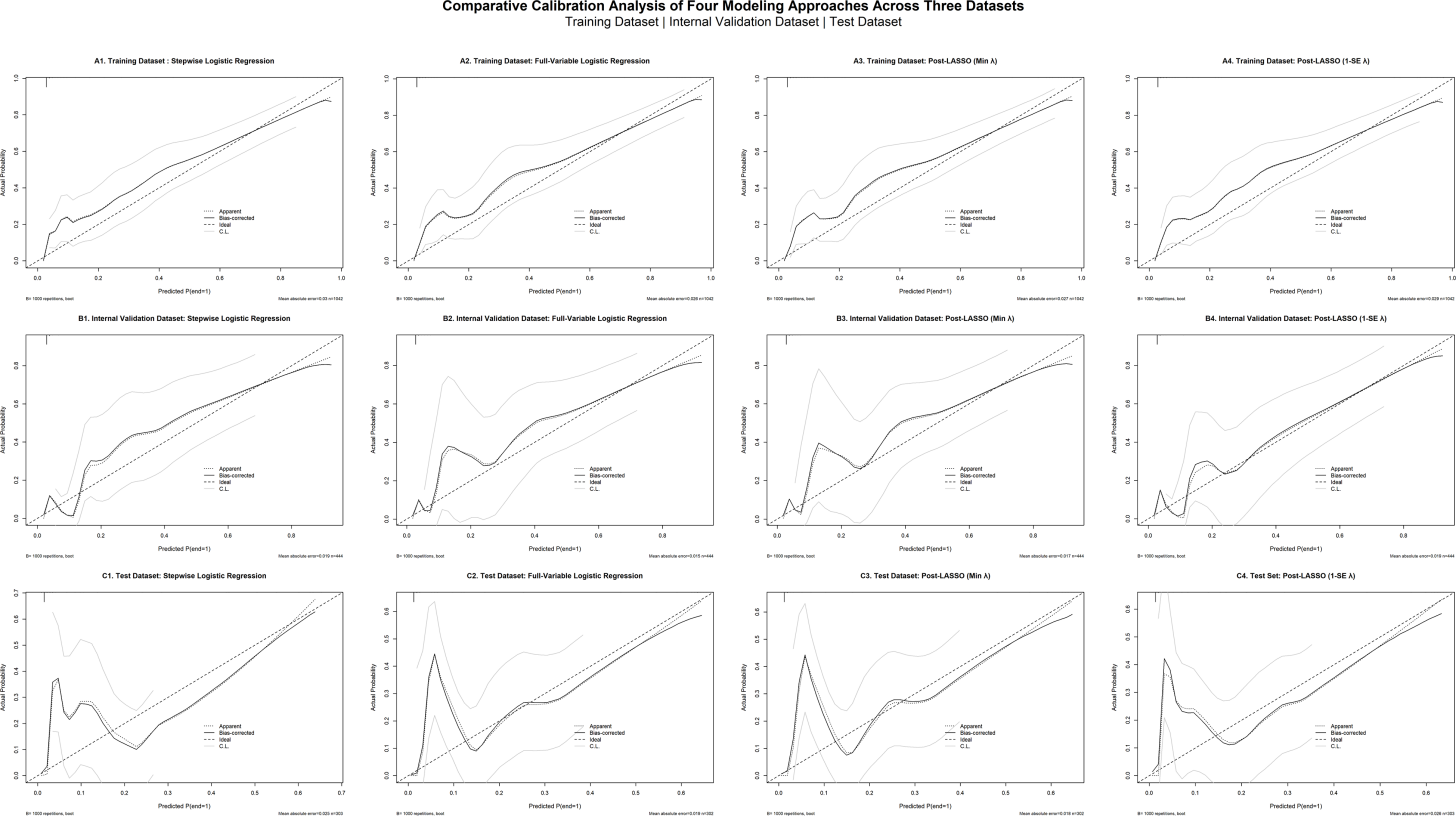
Comparative Calibration Analysis of Four Modeling Approaches Across Three Datasets. **(A1)** Training Dataset: Stepwise Logistic Regression; **(A2)** Training Dataset: Ful-Variable Logistic Regression; **(A3)** Training Dataset PostLASSO (Min λ); **(A4)** Training Dataset.PosLASSO (1-SE λ); **(B1)** Imternal Validation Dataset: Stepwise Logistic Regression; **(B2)** Imteral Validation Dataset Ful-Variable Logistic Regresion; **(B3)** Internal Validation Dataset: Post-LASSO (Min λ); **(B4)** Internal Validation Dataset: Post-LASSO (1-SE λ); **(C1)** Test Dataset: Stepwise Logistic Regression; **(C2)** Test Dataset: Full-Variable Logistic Regression; **(C3)** Test Dataset:Post-LASSO (Min λ); **(C4)** Test Set: Post-LASSO (1-SE λ).

To further evaluate the accuracy of the predicted probabilities, calibration curves were generated to assess the agreement between the estimated and observed probabilities of postoperative complications (POR). In the training cohort, all models demonstrated excellent calibration, with bias-corrected curves closely approximating the ideal diagonal line. The calibration slopes were consistently above 0.81, and the Brier scores remained below 0.042, indicating well-calibrated predictions. In the internal validation cohort, the calibration curves maintained stability, with all slopes exceeding 0.81 and Brier scores below 0.044. However, in the test dataset, a more pronounced decline in calibration performance was observed, particularly in the lower probability range, indicating a notable overestimation of risk. This finding suggests that the models’ calibration substantially deteriorated when applied to the test dataset. The calibration metrics showed minimal variation across the different models, indicating comparable performance in terms of probability estimation (**Figure 3**, **Supplementary Table 7**).

### 3.7 Model selection and nomogram construction

**Figure 4:**
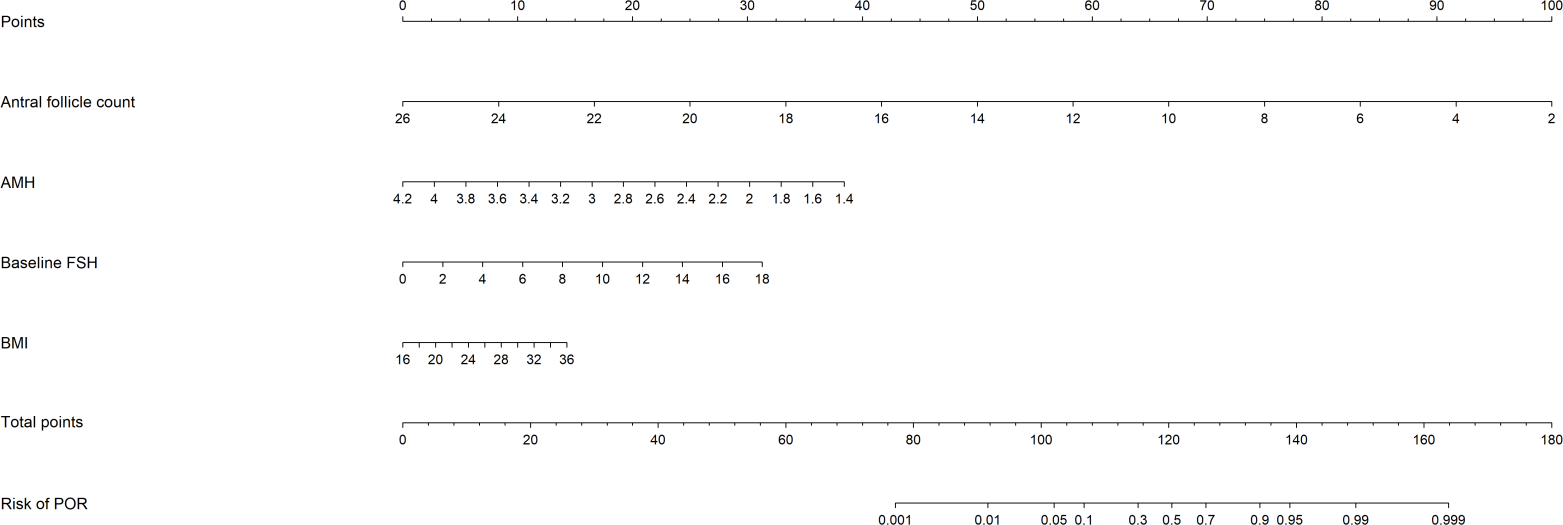
Nomogram for predicting the risk of poor ovarian response.

Based on the comprehensive model performance evaluation and guided by Occam’s razor principle, the stepwise logistic regression was ultimately selected as the final predictive model. This choice ensures that the model not only maintains high predictive accuracy but also exhibits strong generalizability and enhanced practicality. To facilitate personalized risk assessment and optimize clinical decision-making, we developed a nomogram based on the aforementioned model, which incorporated AMH, basal FSH, BMI, and antral follicle count as predictive variables (**Figure 4**). This graphical tool enables the estimation of an individual’s risk of POR by assigning a score to each predictor, with the total score translating into a probability of POR occurrence. The nomogram provides a simple yet effective method for clinicians to personalize patient risk assessments and guide treatment decisions.

## 4 Discussion

In clinical practice, assisted reproductive technology (ART) has been increasingly utilized worldwide (20). ART now has become a cornerstone of infertility management, with clinical practice patterns evolving significantly over the past decade (21). Given the variability in treatment outcomes, predicting ART success has emerged as a critical component of clinical decision-making, enabling personalized treatment planning and patient counseling (22, 23). In this study, we developed and validated a predictive model for poor ovarian response (POR) in patients undergoing assisted reproductive technology (ART). The model integrates clinical variables and ovarian reserve markers, including body mass index (BMI), antral follicle count (AFC), anti-Müllerian hormone (AMH), and basal follicle-stimulating hormone (FSH). Our findings underscore the crucial role of these variables in predicting POR and demonstrate that integrating multiple clinical factors improves the accuracy of ovarian response predictions. The stepwise logistic regression model, chosen based on its balance between predictive accuracy and model simplicity, outperformed traditional regression models, showing superior predictive power, stability, and clinical applicability, making it a reliable tool for clinical decision-making.

Body mass index (BMI) has been well established as a key factor influencing ovarian reserve and assisted reproductive technology (ART) outcomes. Elevated BMI is associated with diminished ovarian response, which may be attributed to obesity-related disturbances in hormonal regulation, insulin resistance, and ovarian follicular development (9, 24). In our cohort, women in the POR group had a significantly higher BMI, which aligns with previous studies linking obesity and advanced maternal age to poorer ovarian response and ART failure (25). Obesity has been shown to increase inflammatory markers and alter gonadotropin secretion, impairing oocyte maturation and embryo development (26). These findings underscore the importance of considering BMI as a critical component in the clinical evaluation of patients undergoing ART, reinforcing its role as a modifiable risk factor for poor ovarian response.

AFC and AMH are currently regarded as the most sensitive and reliable markers of ovarian reserve, and their roles in ART have been extensively investigated (27). Accumulating evidence indicates that both AFC and AMH are among the most accurate predictors of ovarian response to controlled ovarian hyperstimulation (COH) (28). Given that clinical decisions often need to account for cases where AFC and AMH are discordant, a growing number of studies emphasize that integrating both markers rather than relying on either alone improves the prediction of oocyte yield and supports more personalized ovarian stimulation strategies, particularly in women with low ovarian reserve (29). Therefore, these markers are widely recognized as reliable predictors of ovarian reserve and response to stimulation (30, 31). AMH, in particular, is a highly sensitive marker of ovarian reserve, as it reflects the number of small antral follicles and is not influenced by the menstrual cycle (32, 33). AFC, which counts the number of visible antral follicles on ultrasound, is another strong predictor of ovarian reserve and ART success (34). Our findings corroborate previous studies, confirming that lower AMH and AFC values are associated with poor ovarian response (35, 36).

FSH has also been incorporated into ART prediction models. Previous studies have shown, elevated basal FSH reflects diminished ovarian reserve and reduced granulosa cell responsiveness to exogenous gonadotropins. Chronically high FSH levels accelerate follicular recruitment but also promote premature luteinization and oocyte meiotic resumption, leading to increased oocyte aneuploidy and lower embryo quality (37). Additionally, elevated FSH disrupts intrafollicular hormonal balance, impairs granulosa cell proliferation, and compromises ooplasmic maturation, ultimately reducing fertilization and implantation rates (38).

The performance of the stepwise logistic regression model was evaluated in terms of predictive accuracy and stability. The strength of the stepwise method lies in its ability to identify the most relevant predictors, ensuring that the model remains simple and interpretable, while effectively capturing the key factors associated with poor ovarian response. In our study, the stepwise logistic regression model retained important predictors such as antral follicle count (AFC), anti-Müllerian hormone (AMH), basal follicle-stimulating hormone (FSH), and body mass index (BMI), highlighting its utility in clinical prediction tasks (39). The calibration curves showed good agreement between predicted and observed probabilities of POR in both the training and internal validation sets, indicating that the model is well-calibrated and provides accurate predictions. However, there was a slight overestimation of risk in the test set, which is a common challenge when applying models to new datasets. This discrepancy is likely due to differences in patient populations, and further refinement of the model may be required to improve calibration across diverse cohorts (40). To facilitate the practical application of our model in clinical practice, we developed a nomogram based on the stepwise logistic regression model. Nomograms are widely used in clinical settings for risk prediction, offering a straightforward and visual representation of statistical models that can be easily interpreted by clinicians (41). The nomogram developed in this study enables clinicians to quickly estimate a patient’s risk of POR, aiding in more personalized treatment decisions. The use of nomograms has been shown to improve clinical decision-making, particularly in complex conditions such as ART, where multiple factors must be considered simultaneously.

This study has several potential sources of bias and limitations that should be considered when interpreting the findings. First, the retrospective, single-center nature of the data limits the generalizability of the results to broader, more diverse populations. The exclusion of hyper-responders from the analysis may introduce selection bias, as it removes a specific subgroup that could affect the model’s applicability in real-world clinical settings, where hypo-, normo-, and hyper-responders coexist. Furthermore, the use of a complete-case analysis, while standard in dealing with missing data, may have introduced bias if the missing data were not randomly distributed. Additionally, the lack of external validation in this study further limits the generalizability of the model. While internal validation was performed, the model’s performance may differ when applied to data from other institutions or populations. The model’s reliance on a relatively limited set of predictors, such as AMH and AFC, may also reduce its accuracy and applicability in more complex clinical scenarios with additional variables. These limitations should be considered when applying this nomogram in clinical practice. Finally, the p-values and confidence intervals reported from the final model are likely underestimated (optimistically biased) due to the model selection procedure, and their interpretation warrants caution. Future studies, particularly multi-center prospective trials with broader inclusion criteria and external validation, are necessary to assess the robustness and generalizability of the model in diverse patient populations.

## 5 Conclusion

In conclusion, this study presents a robust and reliable predictive model for POR that integrates clinical and ovarian reserve markers. The stepwise logistic regression model demonstrated strong predictive accuracy and calibration, making it an ideal tool for personalized patient management in ART. The incorporation of the nomogram into clinical practice can enhance decision-making, improve patient outcomes, and provide clinicians with a reliable and interpretable approach to predicting POR. Future studies should focus on expanding the sample size and validating this model in diverse populations, while exploring the incorporation of additional biomarkers to further refine its predictive accuracy. The integration of the nomogram into routine clinical practice may significantly improve decision-making in ART, facilitating more individualized patient care and better treatment outcomes.

## Data Availability

The original contributions presented in the study are included in the article/**Supplementary Material**. Further inquiries can be directed to the corresponding author.
