# Supplementary material for: Development and validation of a clinical prediction model for poor ovarian response in assisted reproductive technology
Source: Front Endocrinol (Lausanne). 2026 Jun 15;17:1732869. doi: 10.3389/fendo.2026.1732869 (PMC13310710; doi:10.3389/fendo.2026.1732869)
Supplement: Supplementary file 1 [file DataSheet1.docx]

**
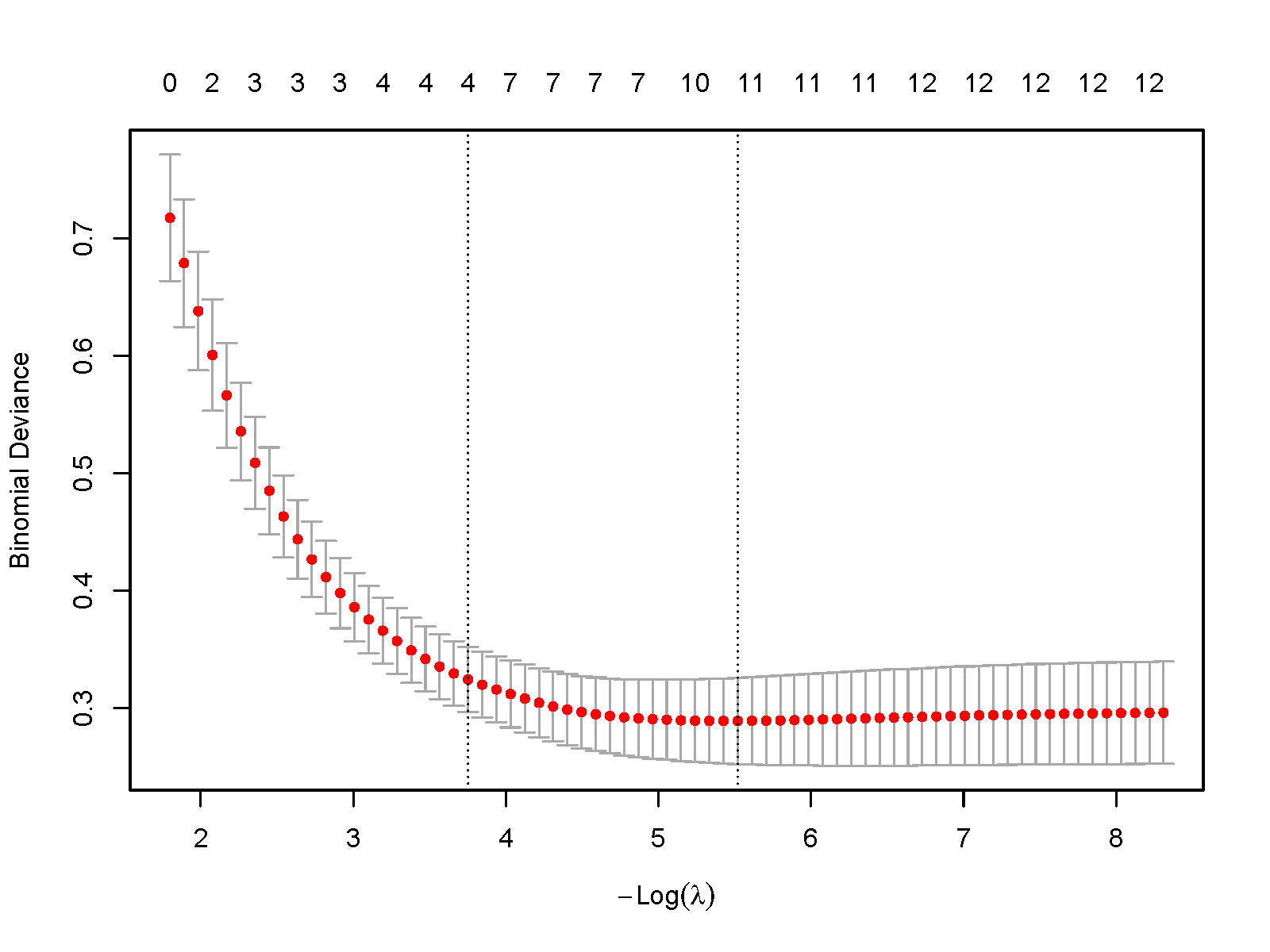
**

**Supplementary Figure 1. The cross-validation curve**

**
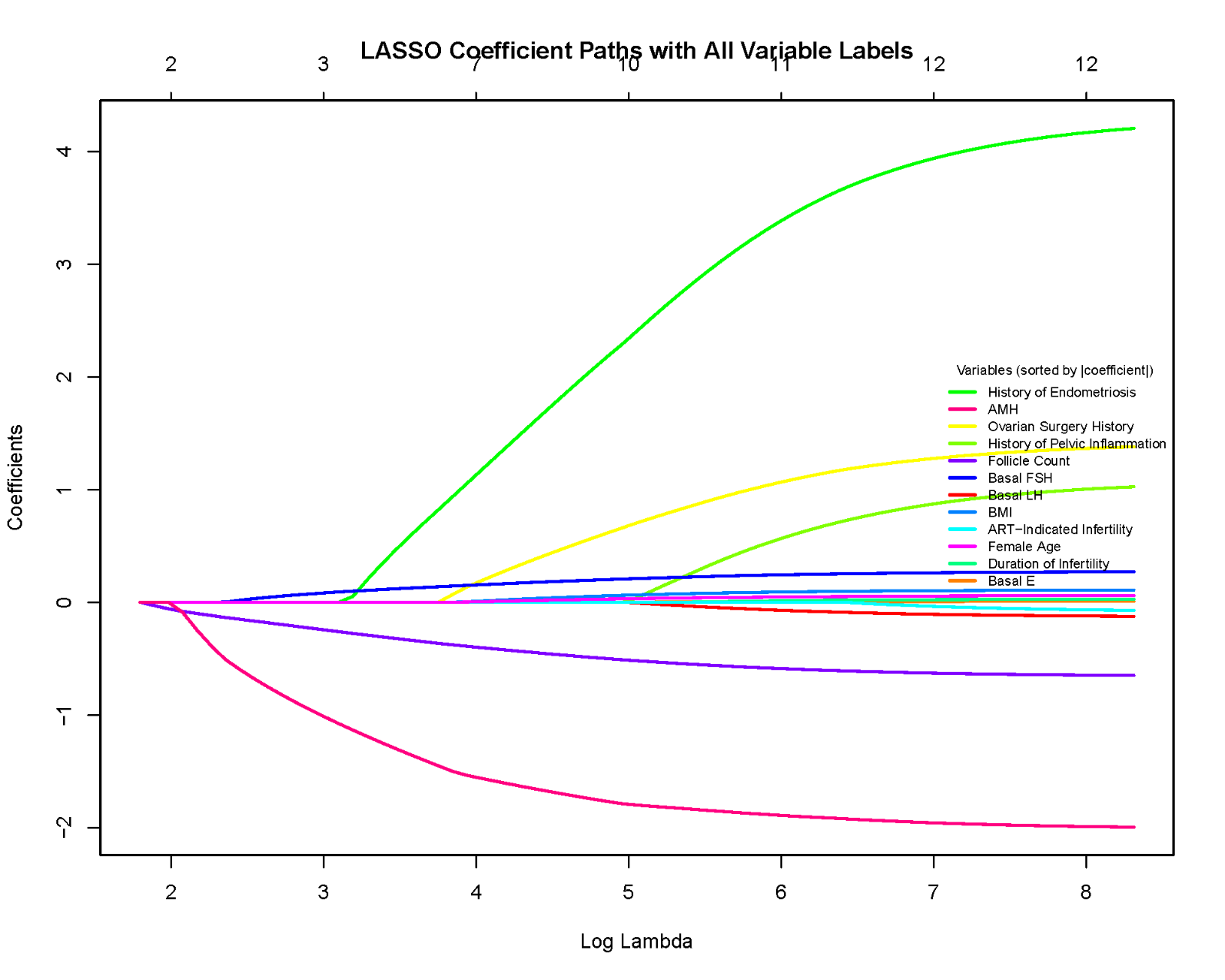
 Supplementary Figure 2. Variable selection pathway analysis**

**Supplementary Table 1: Baseline Characteristics of Patients with Normal and Poor Ovarian Response**

| **Item** | **Missing Cases (n)** | **Missing Data, n (%)** | **Imputation Method** |
| --- | --- | --- | --- |
| Female Age | 0 | 0.00% | - |
| BMI | 0 | 0.00% | - |
| Basal FSH | 4 | 0.27% | 6.65 |
| Basal LH | 0 | 0.00% | - |
| Basal E | 0 | 0.00% | - |
| AMH | 0 | 0.00% | - |
| Ovarian Surgery History | 0 | 0.00% | - |
| History of Pelvic Inflammation | 0 | 0.00% | - |
| History of Endometriosis | 0 | 0.00% | - |
| History of POR | 0 | 0.00% | - |
| Duration of Infertility | 0 | 0.00% | - |
| ART-Indicated Infertility | 0 | 0.00% | - |
| ART-Indicated Infertility | 0 | 0.00% | - |

**Supplementary Table 2: Baseline Characteristics of Patients in Training dataset and Internal validation dataset.**

| **Item** | **Category** | **Total Cohort** | **Normal Ovarian Response** | **Poor Ovarian Response** | **P-value** | **Statistic** |
| --- | --- | --- | --- | --- | --- | --- |
| Female Age |  | 32.10±4.10 | 32.10±4.10 | 32.09±4.10 | 0.9381 | 0.08 |
| Duration of Infertility |  | 3.85±2.73 | 3.89±2.72 | 3.75±2.76 | 0.3904 | 0.86 |
| ART-Indicated Infertility | Primary Infertility | 814(54.78) | 569(54.61) | 245(55.18) | 0.8388 | 0.04 |
|  | Secondary Infertility | 672(45.22) | 473(45.39) | 199(44.82) |  |  |
|  | Total | 1486(100.00) | 1042(100.00) | 444(100.00) |  |  |
| BMI |  | 23.84±3.72 | 23.91±3.74 | 23.67±3.69 | 0.2652 | 1.11 |
| Basal FSH |  | 6.83±1.81 | 6.84±1.86 | 6.80±1.69 | 0.7260 | 0.35 |
| Basal LH |  | 4.96±2.31 | 4.97±2.35 | 4.92±2.22 | 0.7312 | 0.34 |
| Basal E |  | 35.68±14.98 | 35.24±14.03 | 36.71±16.97 | 0.0840 | -1.73 |
| Follicle Count |  | 13.14±4.80 | 13.11±4.85 | 13.20±4.68 | 0.7457 | -0.32 |
| AMH |  | 1.68±0.37 | 1.68±0.37 | 1.70±0.37 | 0.3501 | -0.93 |
| Ovarian Surgery History | None | 1465(98.59) | 1029(98.75) | 436(98.20) | 0.4074 | 0.69 |
|  | Yes | 21(1.41) | 13(1.25) | 8(1.80) |  |  |
|  | Total | 1486(100.00) | 1042(100.00) | 444(100.00) |  |  |
| History of Pelvic Inflammation | None | 1468(98.79) | 1032(99.04) | 436(98.20) | 0.1744 | 1.85 |
|  | Yes | 18(1.21) | 10(0.96) | 8(1.80) |  |  |
|  | Total | 1486(100.00) | 1042(100.00) | 444(100.00) |  |  |
| History of Endometriosis | None | 1467(98.72) | 1029(98.75) | 438(98.65) | 0.8706 | 0.03 |
|  | Yes | 19(1.28) | 13(1.25) | 6(1.35) |  |  |
|  | Total | 1486(100.00) | 1042(100.00) | 444(100.00) |  |  |
| Oocyte Collection Low Response | Normal Response | 1324(89.10) | 921(88.39) | 403(90.77) | 0.1782 | 1.81 |
|  | Low Response | 162(10.90) | 121(11.61) | 41(9.23) |  |  |
|  | Total | 1486(100.00) | 1042(100.00) | 444(100.00) |  |  |

**Supplementary Table 3: Full-Variable Logistic Regression for Predicting Poor Ovarian Response**

| **Item** | **Univariate OR (95% CI)** | **Univariate P-value** | **Multivariate OR (95% CI)** | **Multivariate P-value** |
| --- | --- | --- | --- | --- |
| Age | 1.22(1.16-1.28) | <.0001 | 1.05(0.98-1.13) | 0.1347 |
| AMH | 0.00(0.00-0.01) | <.0001 | 0.14(0.04-0.42) | 0.0006 |
| ART-Indicated Infertility | 1.70(1.16-2.49) | 0.0067 | 0.92(0.48-1.75) | 0.7923 |
| Basal E | 1.01(1.00-1.02) | 0.1327 | 1.01(0.99-1.03) | 0.4698 |
| Basal FSH | 1.94(1.71-2.19) | <.0001 | 1.31(1.12-1.53) | 0.0009 |
| Basal LH | 0.85(0.77-0.95) | 0.0031 | 0.89(0.75-1.06) | 0.2043 |
| BMI | 1.07(1.02-1.12) | 0.0062 | 1.11(1.02-1.21) | 0.0157 |
| Duration of Infertility | 1.01(0.94-1.08) | 0.755 | 1.03(0.93-1.15) | 0.5594 |
| Antral Follicle Count | 0.44(0.39-0.51) | <.0001 | 0.52(0.45-0.60) | <.0001 |
| History of Pelvic Inflammation | 3.32(0.85-13.01) | 0.0851 | 2.78(0.13-59.26) | 0.5121 |
| Ovarian Surgery History | 12.97(4.17-40.32) | <.0001 | 3.87(0.32-47.32) | 0.2889 |

**Supplementary Table 4: Post-LASSO Refit Logistic Regression Model (One Standard Error of Minimum Lambda) for Predicting Poor Ovarian Response**

| **Item** | **Univariate OR (95% CI)** | **Univariate P-value** | **Multivariate OR (95% CI)** | **Multivariate P-value** |
| --- | --- | --- | --- | --- |
| Age | 1.22(1.16-1.28) | <.0001 | 1.05(0.98-1.13) | 0.1361 |
| AMH | 0.00(0.00-0.01) | <.0001 | 0.13(0.04-0.39) | 0.0003 |
| Basal FSH | 1.94(1.71-2.19) | <.0001 | 1.30(1.11-1.52) | 0.0009 |
| BMI | 1.07(1.02-1.12) | 0.0062 | 1.12(1.03-1.22) | 0.0100 |
| Antral Follicle Count | 0.44(0.39-0.51) | <.0001 | 0.52(0.45-0.60) | <.0001 |
| Ovarian Surgery History | 12.97(4.17-40.32) | <.0001 | 3.90(0.31-48.60) | 0.2898 |
| ART-Indicated Infertility | 1.70(1.16-2.49) | 0.0067 |  |  |
| Basal E | 1.01(1.00-1.02) | 0.1327 |  |  |
| Basal LH | 0.85(0.77-0.95) | 0.0031 |  |  |
| Duration of Infertility | 1.01(0.94-1.08) | 0.7550 |  |  |
| History of Pelvic Inflammation | 3.32(0.85-13.01) | 0.0851 |  | . |

**Supplementary Table 5: Post-LASSO Refit Logistic Regression Model (Minimum Lambda) for Predicting Poor Ovarian Response**

| **Item** | **Univariate OR (95% CI)** | **Univariate P-value** | **Multivariate OR (95% CI)** | **Multivariate P-value** |
| --- | --- | --- | --- | --- |
| Age | 1.22(1.16-1.28) | <.0001 | 1.05(0.98-1.13) | 0.1404 |
| AMH | 0.00(0.00-0.01) | <.0001 | 0.14(0.05-0.43) | 0.0006 |
| Basal E | 1.01(1.00-1.02) | 0.1327 | 1.01(0.99-1.03) | 0.4534 |
| Basal FSH | 1.94(1.71-2.19) | <.0001 | 1.31(1.12-1.53) | 0.0008 |
| Basal LH | 0.85(0.77-0.95) | 0.0031 | 0.89(0.75-1.06) | 0.2058 |
| BMI | 1.07(1.02-1.12) | 0.0062 | 1.11(1.02-1.21) | 0.0156 |
| Duration of Infertility | 1.01(0.94-1.08) | 0.755 | 1.04(0.93-1.15) | 0.4983 |
| Antral Follicle Count | 0.44(0.39-0.51) | <.0001 | 0.52(0.45-0.60) | <.0001 |
| History of Pelvic Inflammation | 3.32(0.85-13.01) | 0.0851 | 2.81(0.13-61.96) | 0.5132 |
| Ovarian Surgery History | 12.97(4.17-40.32) | <.0001 | 4.00(0.33-48.97) | 0.2787 |
| ART-Indicated Infertility | 1.70(1.16-2.49) | 0.0067 |  | . |

**Supplementary Table 6: Predictive performance of the models as measured by the area under the ROC curve (AUC).**

| **Data** | **Model** | **AUC** | **95% CI** | **P value vs Model 1** | **P value vs Model 2** | **P value vs Model 3** | **P value vs Model 4** |  |
| --- | --- | --- | --- | --- | --- | --- | --- | --- |
| Training dataset | Model 1 | 0.9726 | 0.9628 – 0.9824 | - | 0.7728 | 0.8334 | 0.1475 |  |
| Training dataset | Model 2 | 0.9730 | 0.9630 – 0.9830 | - | - | 0.4585 | 0.2395 |  |
| Training dataset | Model 3 | 0.9729 | 0.9629 – 0.9829 | - | - | - | 0.2642 |  |
| Training dataset | Model 4 | 0.9704 | 0.9600 – 0.9809 | - | - | - | - |  |
| Internal validation dataset | Model 1 | 0.9538 | 0.9289 – 0.9786 | - | 0.3163 | 0.3235 | 0.0235 |  |
| Internal validation dataset | Model 2 | 0.9482 | 0.9171 – 0.9793 | - | - | 0.8946 | 0.4836 |  |
| Internal validation dataset | Model 3 | 0.9481 | 0.9169 – 0.9794 | - | - | - | 0.4913 |  |
| Internal validation dataset | Model 4 | 0.9424 | 0.9135 – 0.9713 | - | - | - | - |  |
| Test dataset | Model 1 | 0.9585 | 0.9351 – 0.9820 | - | 0.7577 | 0.8015 | 0.1117 |  |
| Test dataset | Model 2 | 0.9592 | 0.9359 – 0.9824 | - | - | 0.7252 | 0.1606 |  |
| Test dataset | Model 3 | 0.9591 | 0.9358 – 0.9823 | - | - | - | 0.1721 |  |
| Test dataset | Model 4 | 0.9525 | 0.9261 – 0.9789 | - | - | - | - |  |

Model 1: Stepwise Logistic Regression

Model 2: Full-Variable Logistic Regression

Model 3: Post-LASSO Refit Logistic Regression Model (Minimum Lambda)

Model 4: Post-LASSO Refit Logistic Regression Model (One Standard Error of Minimum Lambda)

**Supplementary Table 7: Model calibration statistics and Brier score.**

| **Data** | **Model** | **Slope** | **95% CI** | **Intercept** | **Brier score** |  |
| --- | --- | --- | --- | --- | --- | --- |
| Training dataset | Model 1 | 0.8272 | 0.8008-0.8536 | 0.1245 | 0.0402 |  |
| Training dataset | Model 2 | 0.8365 | 0.8050-0.8680 | 0.1178 | 0.0391 |  |
| Training dataset | Model 3 | 0.8310 | 0.7978-0.8641 | 0.1237 | 0.0391 |  |
| Training dataset | Model 4 | 0.8262 | 0.7815-0.8710 | 0.1300 | 0.0412 |  |
| Internal validation dataset | Model 1 | 0.9200 | 0.8546-0.9854 | 0.0697 | 0.0430 |  |
| Internal validation dataset | Model 2 | 0.8448 | 0.7859-0.9037 | 0.1090 | 0.0427 |  |
| Internal validation dataset | Model 3 | 0.8446 | 0.7854-0.9039 | 0.1092 | 0.0428 |  |
| Internal validation dataset | Model 4 | 0.8788 | 0.7971-0.9604 | 0.1002 | 0.0474 |  |
| Test dataset | Model 1 | 0.7007 | 0.5518-0.8497 | 0.1028 | 0.0793 |  |
| Test dataset | Model 2 | 0.7794 | 0.6339-0.9248 | 0.0675 | 0.0794 |  |
| Test dataset | Model 3 | 0.7939 | 0.6516-0.9362 | 0.0616 | 0.0794 |  |
| Test dataset | Model 4 | 0.7911 | 0.6467-0.9356 | 0.0681 | 0.0787 |  |

Model 1: Stepwise Logistic Regression

Model 2: Full-Variable Logistic Regression

Model 3: Post-LASSO Refit Logistic Regression Model (Minimum Lambda)

Model 4: Post-LASSO Refit Logistic Regression Model (One Standard Error of Minimum Lambda)
